# sptotal: an R package for predicting totals and weighted sums from spatial data

**DOI:** 10.21105/joss.05363

**Published:** 2023-05-24

**Authors:** Matt Higham, Jay Ver Hoef, Bryce Frank, Michael Dumelle

**Affiliations:** 1 St. Lawrence University; 2 National Oceanic and Atmospheric Administration; 3 Bureau of Land Management; 4 United States Environmental Protection Agency

## Abstract

In ecological or environmental surveys, it is often desired to predict the mean or total of a variable in some finite region. However, because of time and money constraints, sampling the entire region is often unfeasible. The purpose of the sptotal R package is to provide software that gives a prediction for a quantity of interest, such as a total, and an associated standard error for the prediction. The predictor, referred to as the Finite-Population-Block-Kriging (FPBK) predictor in the literature (J. M. Ver Hoef, 2008), incorporates possible spatial correlation in the data and also incorporates an appropriate variance reduction for sampling from a finite population.

In the remainder of the paper, we give an overview of both the background of the method and of the sptotal package.

## Statement of Need

sptotal provides an implementation of the Finite Population Block Kriging (FPBK) methods developed in J. Ver Hoef (2002) and J. M. Ver Hoef (2008). Next we provide a short overview of FPBK.

Suppose that we have a response variable $Y\left(s_{i}\right),\;i=1,\;2,\ldots ,N$, where the vector $s_{i}$ contains the coordinates for the *i*^*th*^ spatial location and N is a finite number of spatial locations. Then y, an N-length column vector of the $Y\left(s_{i}\right)$, can be modeled with a spatial linear model

(1) y=Xβ+ϵ,

where X is a design matrix for the fixed effects and β is a parameter vector of fixed effects. The vector of random errors follows a multivariate normal distribution with a mean vector of **0** and a covariance of

(2) $$var(\epsilon )=\sigma^{2}R+\tau^{2}I,$$

where $\sigma^{2}$ is the spatial dependent error variance (commonly called the partial sill), $\tau^{2}$ is the spatial independent error variance (commonly called the nugget), and I is the identity matrix. The *i*^*th*^ row and *j*^*th*^ column of the N×N spatial correlation matrix R contains the correlation between the random error of the *i*^*th*^ spatial location, $\epsilon_{i}$, and the random error of the *j*^*th*^ spatial location, $\epsilon_{j}$. A common model used to generate R is the exponential correlation function (Cressie, 2015).

FPBK predicts some linear function of the response, $f(y)=b^{\prime }y$, where b is an N-length column vector of weights. A common vector of weights is a vector of 1’s so that the resulting prediction is for the total abundance across all sites. If only some of the values in y are observed, then the sptotal package can be used to find the the Best Linear Unbiased Predictor (BLUP) for $b^{\prime }y$, referred to as the FPBK predictor, along with its prediction variance.

The primary functions in the sptotal package are described in the following section. In short, the FPBK method is implemented in sptotal’s predict() generic function, which is used on a spatial model that is fit with sptotal::slmfit().

## Package Methods

Before discussing comparable methods and R packages, we show how the main functions in sptotal can be used on a real data set to predict total abundance of moose in a region of Alaska. We use the AKmoose_df data in the sptotal package, provided by the Alaska Department of Fish and Game.


library(sptotal)
data(“AKmoose_df”)


The data contains a response variable total, x-coordinate centroid variable x, y-coordinate centroid variable y, and covariates elev_mean (the elevation) and strat (a stratification variable). There are a total of 860 rows of unique spatial locations. Locations that were not surveyed have an NA value for total.

The two primary functions in sptotal are slmfit(), which fits a spatial linear model, and predict.slmfit(), which uses FPBK to predict a quantity of interest (such as a mean or total) using a fitted slmfit object. slmfit() has required arguments formula, data, xcoordcol, and ycoordcol. If data is a simple features object from the sf (Pebesma & others, 2018) package, then xcoordcol and ycoordcol are not required. The CorModel argument is the correlation model used for the errors.


moose_mod <- slmfit(total ~ elev_mean + strat, data = AKmoose_df,
                          xcoordcol = “x”, ycoordcol = “y”,
                          CorModel = “Exponential”)
summary(moose_mod)
##
## Call:
## total ~ elev_mean + strat
##
## Residuals:
##    Min     1Q Median     3Q     Max
## −4.695 −3.768 −1.304 1.111 35.816
##
## Coefficients:
##             Estimate Std. Error t value Pr(>|t|)
## (Intercept) 0.700203    2.128817    0.329    0.74254 
## elev_mean   0.004479    0.006620    0.677    0.49944
## stratM      2.586271    0.868335    2.978    0.00323 **
## ---
## Signif. codes: 0 ‘***’ 0.001 ‘**’ 0.01 ‘*’ 0.05 ‘.’ 0.1 ‘ ‘ 1
##
## Covariance Parameters:
##              Exponential Model
## Nugget                29.71177
## Partial Sill           8.04658
## Range                 33.65766
##
## Generalized R-squared: 0.03966569


With the summary() generic, we obtain output similar to the summary output of a linear model fit with lm(), as well as a table of fitted covariance parameter estimates. Next, we use predict() to implement FPBK and obtain a prediction for the total abundance across all spatial locations, along with a standard error for the prediction. By default, predict() gives a prediction for total abundance, though the default can be modified by specifying a column of prediction weights for the vector b with the wtscol argument.


predict(moose_mod)
## Prediction Info:
##       Prediction     SE 90% LB 90% UB
## total       1610 413.2    930.1   2290
##       Numb. Sites Sampled Total Numb. Sites Total Observed Average Density
## total                 218               860            742           3.404


The output of printed predict() gives a table of prediction information, including the Prediction (a total abundance of 1610 moose, in this example), the SE (Standard Error) of the prediction, and bounds for a prediction interval (with a nominal level of 90% by default). Additionally, some summary information about the data set used is given.

sptotal also provides many helper generic functions for spatial linear models. The structure of the arguments and of the output of these generics often mirrors that of the generics used for base R linear models fit with lm(). Examples (applied to the moose_mod object) include AIC(moose_mod), coef(moose_mod), fitted(moose_mod), plot(moose_mod), and residuals(moose_mod).

### Comparable Methods and Related Work

Design-based analysis and k-nearest neighbors (Fix, 1985) are two approaches that can be used to compute a mean or total in a finite population. Dumelle, Higham, Ver Hoef, Olsen, & Madsen (2022) provide an overview of design-based spatial analysis and FPBK, showing that FPBK often outperforms the design-based analysis. J. M. Ver Hoef & Temesgen (2013) show that FPBK often outperforms k-nearest-neighbors and highlight that quantifying uncertainty is much more challenging with k-nearest-neighbors.

Note that there are many spatial packages in R that can be used to predict values at unobserved locations, including gstat (Pebesma, 2004), geoR (Ribeiro Jr, Diggle, Schlather, Bivand, & Ripley, 2020), and spmodel (Dumelle, Higham, & Ver Hoef, 2023), among others. What sptotal contributes is the ability to obtain the appropriate variance of a linear combination of predicted values that incorporates a variance reduction when sampling from a finite number of sampling units.

### Past and Ongoing Research Projects

Dumelle et al. (2022) used the sptotal package to compare model-based and design-based approaches for analysis of spatial data. Currently, a Shiny app is in development at the Alaska Department of Fish and Game that uses sptotal to predict abundance from moose surveys conducted in Alaska.
